# High Serum Levels of Otolin-1 in Patients With Benign Paroxysmal Positional Vertigo Predict Recurrence

**DOI:** 10.3389/fneur.2022.841677

**Published:** 2022-03-14

**Authors:** Zhenyi Fan, Zhizhou Hu, Weiwei Han, Xiaoxiong Lu, Xiaoxia Liu, Min Zhou, Wang Yan, Yunqin Wu

**Affiliations:** ^1^Department of Neurology, Hwa Mei Hospital, University of Chinese Academy of Science, Ningbo, China; ^2^Ningbo Institute of Life and Health Industry, University of Chinese Academy of Science, Ningbo, China; ^3^Department of Neurology, Longyan First Affiliated Hospital, Fujian Medical University, Longyan, China; ^4^Department of Rehabilitation, Hwa Mei Hospital, University of Chinese Academy of Science, Ningbo, China

**Keywords:** benign paroxysmal positional vertigo, otolin-1, recurrence, canalith repositioning procedure, biomarker

## Abstract

**Background:**

Otolin-1 is an inner ear-specific protein that is exclusively expressed in otoconia and vestibule and cochlea cells. Recent investigations reported that otolin-1 can cross the blood-labyrinthine barrier and that the levels in serum well-reflected otolith status. Serum otolin-1 levels in patients with benign paroxysmal positional vertigo (BPPV) are significantly elevated compared with healthy controls. We aimed to explore whether otolin-1 can also serve as a biomarker for predicting BPPV recurrence.

**Method:**

Patients at our institution with new-onset of idiopathic BPPV between May, 2017 and May, 2018 were recruited and followed up for 2 years. All demographic data of the patients were collected, and serum levels of otolin-1 and other laboratory indicators were measured and compared according to the recurrence status.

**Results:**

A total of 74 patients, who met the inclusion criteria were enrolled in this study, of which 27 (36.5%) patients had suffered one or more episodes of recurrence after undergoing canal repositioning treatments during the study. The serum levels of otolin-1 in patients with recurrent BPPV were significantly higher than those in patients without recurrent BPPV (363.9 vs. 309.8 pg/ml, *p* = 0.001). In multivariate analysis comparing the second to fourth quartiles (Q2–Q4) against the first quartile (Q1) of otolin-1, the level of otolin-1 in Q4 could significantly predict BPPV recurrence, and the odds ratio (OR) was elevated by approximately 812% (OR = 9.12; 95% confidence interval [CI]: 1.44–57.9; *p* = 0.019).

**Conclusion:**

High serum levels of otolin-1 were associated with an increased risk of BPPV recurrence, and further investigation is required to confirm this association and clarify the exact mechanism.

## Introduction

Benign paroxysmal positional vertigo (BPPV) is the most common cause of peripheral vertigo and affects nearly 17% of patients with dizziness or vertigo. The typical clinical presentation of transient vertigo attacks with characteristic nystagmus is provoked by head position changes (1, 2). Canalith repositioning manoeuvers (CRMs) are the first-line treatments for BPPV, and symptoms can be relieved in more than 95% of cases (3). Additionally, BPPV cases will resolve spontaneously within a few weeks. However, BPPV sometimes recurs with a reported recurrence rate ranging from 7 to 50%, depending on the clinical setting, which results in anxiety and poor quality of life in patients (4, 5). Several studies have explored the risk factor-associated recurrence of BPPV, but the conclusions are conflicting (6, 7). Therefore, exploring the potential markers that predict the recurrence of BPPV may have an important value for the integrated management of BPPV.

Otolin-1 is a scaffolding glycoprotein whose messenger RNA (mRNA) is exclusively expressed in otoconia and cells of semicircular canal cristae, organ of Corti, and marginal cells of the stria vascularis (8–10). Since Parham et al. first reported that otolin-1 can be detected in peripheral blood, some investigations have been carried out and confirmed that serum levels of otolin-1 in patients with BPPV were significantly elevated compared with those in healthy controls (11–14). In addition, the levels of otolin-1 in healthy people increase with age, which is consistent with the age-related demineralization of otoconia and the increased prevalence of BPPV with age (15).

All these studies provided proof of concept for using the inner specific protein otolin-1 as a circulatory biomarker for otoconia degeneration. We speculate that the more severe the otoconia degeneration is, the higher the level of otolin-1. In people with higher levels of otolin-1, otoconia might be more easily exfoliated from the maculae and tends to cause vertigo attacks (16). To date, no studies in the literature have corroborated the recurrence of BPPV with the levels of inner-specific proteins. In this study, we took an initial step to further investigate whether the serum level of otolin-1 can serve as a biomarker for predicting BPPV recurrence events.

## Methods

This study was approved by our institutional review board (protocol number KY-2017-014) and performed in accordance with the tenets of the Declaration of Helsinki. Informed consent was obtained from all participants. Patients who were diagnosed with *de novo* idiopathic BPPV at the Department of Neurology and Emergency, Hwa Mei Hospital, University of Chinese Academy of Science from May 2017 to May 2018 were included. The detailed recruitment and exclusion criteria were described in previous study (12). Since informed consent was obtained, morning fasting blood samples were collected, routine laboratory indexes were assessed, and the remaining serum was stored at −80°C until further analysis. Serum levels of otolin-1 were assessed by a human enzyme-linked immunosorbent assay kit (category number: QY-E03713; QAYEE-BIO, Shanghai, China).

Patients were treated with appropriate CRM and re-evaluated after 7 days until the symptoms and nystagmus were absent. Once the patient's vertigo attack occurred, he or she was asked to return to our hospital to be re-examined. If the interval between the onset of two symptoms was more than 1 month, it was defined as a recurrence. Patients were followed up regularly by telephone or as outpatient at 1, 3, 6, 12, and 24 months. All the data that were recorded included age, sex, lifestyle habits, ongoing health problems, medication history, affected semicircular canal, onset time, interval between blood collection and symptom onset, recurrence rate, and laboratory indicators.

### Statistical Analysis

Statistical analysis was performed using SPSS 22.0 (SPSS Inc., Chicago, IL, USA). Continuous variables following normal distributions are expressed as mean ± standard deviation, and those that did not follow a normal distribution are expressed as the median and interquartile range (IQR). Categorical variables are presented as the number of cases and percentage. Comparison between categorical variables was performed using a *t*-test, the chi-square test, Fisher's test, or Mann-Whitney *U*-test. Multivariable logistic regression was performed to evaluate the recurrence risk factors for BPPV patients. Multivariate analysis was used to assess recurrent BPPV according to otolin-1 quartiles (the lowest quartile [Q1] was used as the reference). A receiver operating characteristic (ROC) curve was generated to evaluate the ability of serum otolin-1 levels to predict recurrent BPPV and no recurrent BPPV. Values of *p* < 0.05 were considered statistically significant.

## Results

### Demographics and Clinical Characteristics of the Subjects

A total of 78 patients diagnosed with *de novo* idiopathic BPPV were included in the study, while 4 patients were eliminated from the analysis due to loss during the follow-up. There were 27 (36.5%) patients who experienced recurrence after CRM treatment, of whom 18 patients (66.7%) suffered one episode of recurrence, while 7 patients (25.9%) suffered two episodes of recurrence and 2 patients (7.4%) suffered three or more episodes of recurrence during the follow-up. The general demographics of these patients are compared in Table 1 according to the number of recurrence episodes.

**Table 1:** Basic demographic characteristics of the subjects.

	**Nonrecurrent group (47)**	**Recurrence group (27)**	**T/Z**	***P***
Age (years)	61.8 ± 11.8	65.5 ± 8.4	−1.415	0.161
Sex (F/M)	31/16	17/10	0.067	0.795
BMI (kg/m2)	23.42 ± 3.2	23.6 ± 3.5	−0.221	0.826
Hypertension [n (%)]	19 (40.4%)	13 (48.1%)	0.417	0.519
Diabetes [n (%)]	8 (17.2%)	4 (14.8%)		0.806^*^
Smoking [n (%)]	8 (17.2%)	5 (18.5%)	0.027	0.871
Drinking [n (%)]	9 (19.1%)	4 (14.8%)		0.757^*^
Time from symptom onset to blood collection (D)	3.55 (1.7–6.5)	4.5 (1.6–7.6)	−0.320	0.749
PSCC: LSCC	31/16	16/11	0.332	0.564

*F, female; M, male; PSCC, posterior semicircular canal; LSCC, lateral semicircular canal. Values are expressed as n (%), mean ± SD or median (interquartile range). P values were calculated using an independent t test, the chi-squared test, Fisher's test or the nonparametric Mann–Whitney U-test*.

* *Fisher's test. P < 0.05 were considered significant*.

### Recurrence Risk Factor Analysis

**Figure 1:**
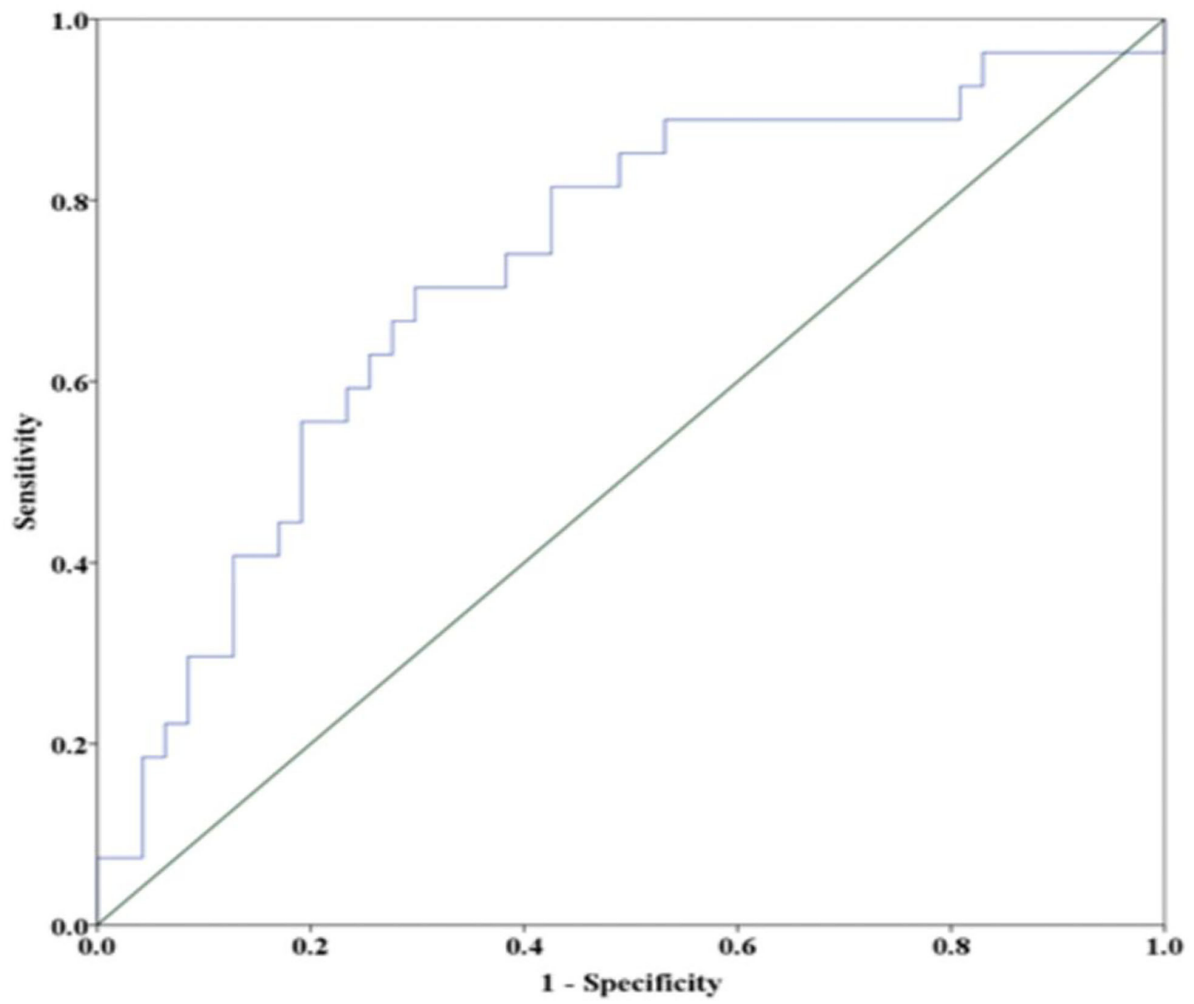
Receiver operator characteristic analysis of otolin-1 for predicting the recurrence BPPV. A cut off value of 334.7 pg/ml provides a sensitivity of 70.4%, a specificity of 74.5%, and an area under the curve of 0.724 (95% CI = 0.602–0.847).

There were no statistical differences in the age distribution, sex ratio, body mass index, lifestyle habits, clinical history, time from symptom onset to blood collection, types of semicircular canal involved, or laboratory results such as hemoglobin, blood creatinine and urea nitrogen, uric acid, liver function, and lipid profiles between the groups (Table 2). Higher serum levels of otolin-1 were observed in patients with recurrent BPPV than in those patients without recurrence (363.9 [IQR: 318.2–417.9] pg/ml vs. 309.8 [280.1–334.9] pg/ml; *Z* = −3.329; *p* = 0.001). Multiple logistic regression analyses showed that the serum level of otolin-1 was an independent risk factor for the recurrence of BPPV (OR: 1.004, 95% CI: 1.000–1.007; *p* = 0.043) (Supplementary Material). The recurrence rate of BPPV across otolin-1 quartiles, ranges from 16.7% (Q1) to 66.7% (Q4). In multivariate analysis models, comparing the second, third, and fourth quartiles (Q2, Q3, and Q4) of otolin-1 levels against Q1, only the levels of otolin-1 in Q4 were correlated with recurrent BPPV (OR: 9.123; 95% CI: 1.44–57.9; *p* = 0.019) (Table 3). A serum otolin-1 value of 334.7 pg/ml was shown to aid in the prediction of BPPV recurrence, with a sensitivity of 70.4%, a specificity of 74.5%, and an area under the cure of 0.724 (95% CI: 0.602–0.847; Figure 1).

**Table 2:** Biochemical parameters of the subjects.

	**Nonrecurrent group (47)**	**Recurrent group (27)**	**F/Z**	***P***
Haemoglobin (g/l)	133 (120–142)	133 (120–141)	−0.596	0.551
Total protein (g/l)	68.5 ± 6.2	68.7 ± 5.7	−0.157	0.876
Albumin (g/l)	41.9 ± 4.0	42.2 ± 3.3	−0.306	0.760
Creatinine (μmol/l)	61.1 (51.1–72.4)	56.6 (48.5–70.4)	−0.545	0.586
Blood urea nitrogen (mmol/l)	4.68 (4.01–5.88)	4.75 (4.28–5.89)	−0.146	0.884
Uric acid (μmol/l)	286.9 ± 64.1	306.6 ± 69.9	−1.232	0.222
Total cholesterol (mmol/l)	4.68 ± 1.23	4.52 ± 1.43	0.490	0.626
HDL (mmol/l)	1.31 ± 0.33	1.28 ± 0.32	0.354	0.725
LDL (mmol/l)	2.61 ± 0.99	2.46 ± 0.78	0.677	0.501
Triglycerides (mmol/l)	1.36 (0.88–1.81)	1.239 (0.88–1.73)	−0.477	0.633
Otolin-1 (pg/ml)	309.8 (280.1–334.9)	363.9 (318.2–417.9)	−3.329	0.001

*HDL, high-density lipoprotein; LDL, low-density lipoprotein. Values are expressed as n (%), mean ± SD or median (interquartile range). P values were calculated using an independent t test, or the nonparametric Mann-Whitney U-test. P < 0.05 were considered significant*.

**Table 3:** Odds ratios (ORs) for recurrence according to otolin-1 quartiles.

Otolin-1 quartiles*(n)	**Recurrence (n/%)**	**Adjusted OR (95% CI)**	***P***
Q1 (*n* = 18)	3 (16.7%)	Reference	
Q2 (*n* = 19)	5 (26.3%)	2.267 (0.333–15.428)	0.403
Q3 (*n* = 19)	8 (36.8%)	4.563 (0.735–28.338)	0.103
Q4 (*n* = 18)	11 (66.7%)	9.123 (1.439–57.855)	0.019

CI, confidence interval. *Serum levels of otolin-1 in quartile 1 (< 284.97 ng/ml), quartile 2 (284.97–324.56 ng/ml), quartile 3 (324.56–382.13 ng/ml), and quartile 4 (>382.13 ng/ml). Adjusted for age, sex, body mass index, time from symptom onset to blood collection, haemoglobin, total protein, albumin, creatinine, blood urea nitrogen, uric acid, total cholesterol, high-density lipoprotein, low-density lipoprotein and triglycerides.

## Discussion

From a clinical perspective, biomarkers for investigating otologic disorders are very helpful and can enable prediction of disease, therapeutic targeting, and response. Otolin-1, an inner ear-specific protein, is a potential circulatory biomarker candidate for otoconia degeneration. Our study investigated the correlation between the serum level of otolin-1 and recurrence events in patients with BPPV. This study showed the following: 1) Serum levels of otolin-1 in patients with recurrent BPPV were significantly higher than those in patients with non-recurrent BPPV; 2) elevated serum levels of otolin-1 were associated with a higher risk of recurrent BPPV, suggesting that otolin-1 may be a risk factor for the recurrence of BPPV.

Otoconia are dense crystals composed of calcium carbonate and an organic matrix, which are synthesized during embryonic development and completely calcified on the seventh postnatal day. Otoconial degeneration and dislodged otoconia falling into the canal are the leading causes of BPPV (17–19). Dislodged otoconia can also be dissolved in the endolymph, and some matrix proteins may be reabsorbed and released into circulation (20). Due to the limitations of current technology, it is difficult to observe otoconia by radiographic testing in real time and even more difficult to study vital specimens. Thus, it is important to explore potential biomarkers for otoconia-related diseases. Therefore, an increasing number of studies have focused on the metabolism of otoconial matrix proteins.

The otoconia organic matrix is composed of a variety of proteins, the chief constituents of which are otoconin-90 and otolin-1. Otolin-1 is a secreted glycoprotein whose mRNA expression is restricted to the support cells of the vestibular maculae, semicircular canal cristae, and organ of Corti and marginal cells of the stria vascularis (10, 21). Parham et al. first reported that otolin-1 could be detected in serum and that the levels were higher in patients with BPPV than in healthy controls (11). Since BPPV in some of these patients was not in the acute stage, only one-third of the serum otolin-1 values of the patients were higher than those in the control range. Since then, a series of studies have been carried out to explore whether otolin-1 has any value in the diagnosis and prognosis of otolith-related diseases. Dogan et al. (22) reported that the levels of serum otolin-1 were significantly increased in patients who had undergone mastoidectomy. Other studies conducted by Irugu et al. (13) and Yadav et al. (14) showed that the serum levels of otolin-1 in patients with BPPV were significantly higher than those in healthy controls. Recently, Naples et al. (23) in a pilot study evaluated a role for prestin and otolin-1 as biomarkers to differentiate Meniere's disease (MD) from vestibular migraine (VM). Prestin and otolin-1 levels were not significantly elevated in MD patients compared to VM patients. Thus, their role in differentiating MD from VM remains to be elucidated in future studies. Previous studies found that the levels of otolin-1 in peripheral blood increase with age, which is consistent with scanning electron microscopy findings of age related degeneration of otoconia and the increased prevalence of BPPV with aged. The changes in serum levels of otolin-1 reported in these studies reflected the processes of breakdown and degradation of otoconia. Similarly, a study found that otoconin-90 could be detected in peripheral blood and the levels in patients with BPPV were significantly higher than those in healthy controls. Otoconin-90 blood levels showed a high positive correlation with age, reflecting the process of otoconia degradation with age (24). All these studies suggested that otolin-1, an otoconia marker, has great clinical application prospects.

BPPV is a benign disease with a high-resolution rate, although it is prone to relapse, which leads to anxiety and poor quality of life in patients and increases the risk of falling due to imbalance (25, 26). Several studies have attempted to clarify relationships between age, diabetes, hypertension, delayed BPPV treatment using CRM, multiple canal involvement, osteoporosis, vitamin D deficiency, and the recurrence of BPPV, but the risk factor-associated recurrence remains elusive (4, 6, 7). Therefore, studying the risk factors for the recurrence of BPPV is imperative for better relapse prevention. The inner ear-specific protein, otolin-1, served as a candidate biomarker in circulation for the diagnosis of BPPV, but there have been no relevant studies investigating whether the serum level of otolin-1 can predict BPPV recurrence events.

Previously, we conducted a study and reported that the serum levels of otolin-1 in active episodes of patients with BPPV were significantly higher than those in healthy controls (324.55 vs. 259.54 pg/ml, *p* < 0.001), and ROC analysis showed that serum otolin-1 cut off value of 299.45 pg/ml could discriminate patients with BPPV from healthy controls with a sensitivity of 67.9% and a specificity of 72.7% (12). During the two-year follow-up period, 27 (36.5%) patients experienced recurrence after initial CRM treatment, which was consistent with previous studies that reported the recurrence rate (27). We found that the levels of otolin-1 in patients with BPPV relapse were significantly higher than those in patients without relapse, and high levels of otolin-1 were shown to be a risk factor for the recurrence of BPPV. These findings suggest that otolin-1 may also serve as a biomarker for BPPV recurrence and has some clinical value in the prediction of recurrence in BPPV patients.

This study has some limitations. First, it was a small-scale, single-center, observational study with a relatively short follow-up time, and the association between otolin-1 levels in circulation and recurrence of BPPV was only suggested. Second, we measured the serum levels of otolin-1 at only one time point in patients who initially presented with symptoms at the time of recruitment to the study. The last is also the primary limitation of the research. To date, there is no information on otolin-1 temporal evolution during the metabolic process of otoconia, as we cannot obtain inner ear tissues in real time and lack a proper animal model. Therefore, research on otolin-1 as a clinical biomarker for BPPV is still in the preliminary stages. Many studies should be conducted to clarify the metabolic process of otolith matrix proteins, which could provide experimental support for the possibility of utilizing otolin-1 as a biomarker for BPPV.

## Conclusion

Elevated serum levels of otolin-1 were associated with an increased risk of recurrent BPPV, but additional work is needed to establish its value and clarify the exact mechanism.

## Data Availability Statement

The datasets analyzed in this article are available upon request to: wu_yunqin@126.com.

## Ethics Statement

The studies involving human participants were reviewed and approved by Hwa Mei Hospital, University of Chinese Academy of Science review board (Protocol Number KY-2017-014). The patients/participants provided their written informed consent to participate in this study.

## Author Contributions

ZF, ZH, WH, XLu, XLi, MZ, and WY included, followed, and recorded patients data. ZF and ZH checked medical records and wrote the article. YW conceived and led the work. All authors contributed to the article and approved the submitted version.

## Funding

This study was supported by Ningbo Medical Key Discipline (Grant No. B12), Ningbo Natural Science Foundation (Grant No. 202003N4240), and Hwa Mei Foundation (Grant No. 2021HMKY 30).

## Conflict of Interest

The authors declare that the research was conducted in the absence of any commercial or financial relationships that could be construed as a potential conflict of interest.

## Publisher's Note

All claims expressed in this article are solely those of the authors and do not necessarily represent those of their affiliated organizations, or those of the publisher, the editors and the reviewers. Any product that may be evaluated in this article, or claim that may be made by its manufacturer, is not guaranteed or endorsed by the publisher.
