# Supplementary material for: High Serum Levels of Otolin-1 in Patients With Benign Paroxysmal Positional Vertigo Predict Recurrence
Source: Front Neurol. 2022 Mar 14;13:841677. doi: 10.3389/fneur.2022.841677 (PMC8963966; doi:10.3389/fneur.2022.841677)
Supplement: Supplementary file 1 [file Table_1.docx]

Table 1 | Multiple logistic regression analysis to identify independent risk factors for BPPV recurrence

| Variables | OR (95% CI) | P |
| --- | --- | --- |
| otolin-1 | 1.000 – 1.007 | 0.043 |

Adjusted for age, sex, body mass index, time from symptom onset to blood collection, haemoglobin, total protein, albumin, creatinine, blood urea nitrogen, uric acid, total cholesterol, high-density lipoprotein, low-density lipoprotein and triglycerides
